# p53 dosage can impede Kras^G12D^- and Kras^Q61R^-mediated tumorigenesis

**DOI:** 10.1371/journal.pone.0292189

**Published:** 2024-03-28

**Authors:** Özgün Le Roux, Jeffery I. Everitt, Christopher M. Counter

**Affiliations:** 1 Department of Pharmacology & Cancer Biology, Duke University Medical Center, Durham, North Carolina, United States of America; 2 Department of Pathology, Duke University Medical Center, Durham, North Carolina, United States of America; Virginia Commonwealth University, UNITED STATES

## Abstract

Mice engineered with a G12D versus Q61R mutation in Kras exhibited differences in tumorigenesis. Namely, the incidence or grade of oral or forestomach squamous epithelial lesions was more prevalent in the Kras^G12D^ background while hematolymphopoietic disease was more prevalent in the Kras^Q61R^ background. Loss of the *Trp53* gene encoding the tumor suppressor p53 enhances the ability of oncogenic Kras to initiate tumorigenesis in carcinogen and genetic models of lung cancer. Conversley, an extra copy of *Trp53* (*Super p53*) was recently shown to suppress Kras-induced tumorigenesis in a genetic model of this disease. Given this, we evaluated whether an extra copy of *Trp53* would alter tumorigenesis upon global activation of a modified *Kras* allele engineered with either a G12D or Q61R mutation. We report that an increase in p53 dosage significantly reduced the incidence or grade of oral and forestomach squamous tumors induced by either G12D and Q61R-mutant Kras. The incidence of myeloproliferative disease was also significantly reduced with increased p53 dosage in the *Kras*^*Q61R*^ background. Both the percentage of mice with lung tumors and total number of adenomas per animal were unchanged. However, the incidence and grade of peripheral atypical alveolar hyperplasia was significantly decreased in both backgrounds with increased p53 dosage. Finally, the number of foci of bronchioloalveolar hyperplasia per animal significantly increased with increased p53 dosage in the *Kras*^*G12D*^ background. These results suggest that an extra copy of p53 can impede oncogenic Kras driven tumorigenesis in some tissues.

## Introduction

The RAS superfamily of small GTPases is comprised of the genes *KRAS*, *NRAS*, and *HRAS* in humans (*Kras*, *Nras*, and *Hras* in mice) [1]. Activated growth factor receptors are known to lead to the switching of RAS from an inactive GDP-bound to an active GTP-bound state. Once activated, RAS recruits proteins with RAS-binding or -associating domains, thereby activating these proteins to propagate signaling. Hydrolysis of GTP returns RAS to the inactive GDP-bound state, terminating the signaling [2]. However, in a fifth or more of human cancers, point mutations at one of three hotspot sites render RAS constitutively active, which is well established to initiate tumorigenesis [1–4]. Between the three RAS genes, three hotspots, with six possible substitutions there are more than 50 potential oncogenic mutations, yet cancers tend to have a particular bias, or a ‘RAS mutation tropism’, towards an often unique subset of these mutations [5]. Case in point, the most common RAS mutations in lung cancer are G12C/V/D in KRAS [6], but Q61R/K in NRAS in melanoma [7].

Expression of different RAS mutants in mice can also affect the nature of tumors arising. For example, expressing a *Kras*^*G12D*^, but not *Nras*^*G12D*^ allele in the skin of mice leads to melanoma [8]. Three oncogenic *Kras* mutations were more commonly detected in tumors arising in mice infected with an sgRNA library designed to generate all 12 oncogenic mutations at codons G12 and G13 [9]. We recently created Cre-inducible (lox-STOP-lox or LSL) *Kras* alleles encoded by common (com) codons and either of the two biochemically distinct mutations G12D or Q61R. Globally activating these two via Cre expressed from the ubiquitous *Rosa26* locus led to differences in the severity and in some cases even type of tumors arising in a manner specific to mutation type. Namely, the incidence or grade of oral or forestomach squamous epithelial lesions was more prevalent in the Kras^G12D^ background while hematolymphopoietic disease was more prevalent in the Kras^Q61R^ background [10].

The pattern of tumors induced by these two alleles is ostensibly a product of how a normal cell responds to different activation levels or specific mutant oncoproteins. In this regard, oncogenic RAS can both induce proliferation or senescence, the latter of which is mediated in part by the tumor suppressor p53 [11]. Recently, “*super p53*” mice encoding an extra copy of *Trp53* [12] were shown to suppress spontaneous Kras-driven lung and lymphoma tumorigenesis arising in an *LA1-Kras*^*G12D*^ background, but not radiation-induced lymphomas [13]. This prompted us to ask whether an increase in p53 dosage would alter the severity or type of tumors arising between different oncogenic Kras mutations. We thus generated mice with the two experimental genotypes of *Kras*^*LSL-comG12D/+*^;*Super p53* and *Kras*^*LSL-comQ61R/+*^;*Super p53* and the two control genotypes of *Kras*^*LSL-comG12D/+*^ and *Kras*^*LSL-comQ61R/+*^ in the *Rosa26*^*CreERT2/+*^ background, which allows Cre expression in a broad range of tissues [14]. The *Kras*^*LSL*^ alleles were activated by tamoxifen treatment and the mice were analyzed for the presence of hematolymphopoietic, oral and forestomach, and lung tumors, as well as tumors in other organs. We report here that an increase in *p53* dosage could reduce tumorigenesis, although this was not observed in every tissue or with both mutant forms of Kras.

## Results

### The experimental design to explore the influence of p53 dosage on tumorigenesis induced by different Kras mutations

**Fig 1:**
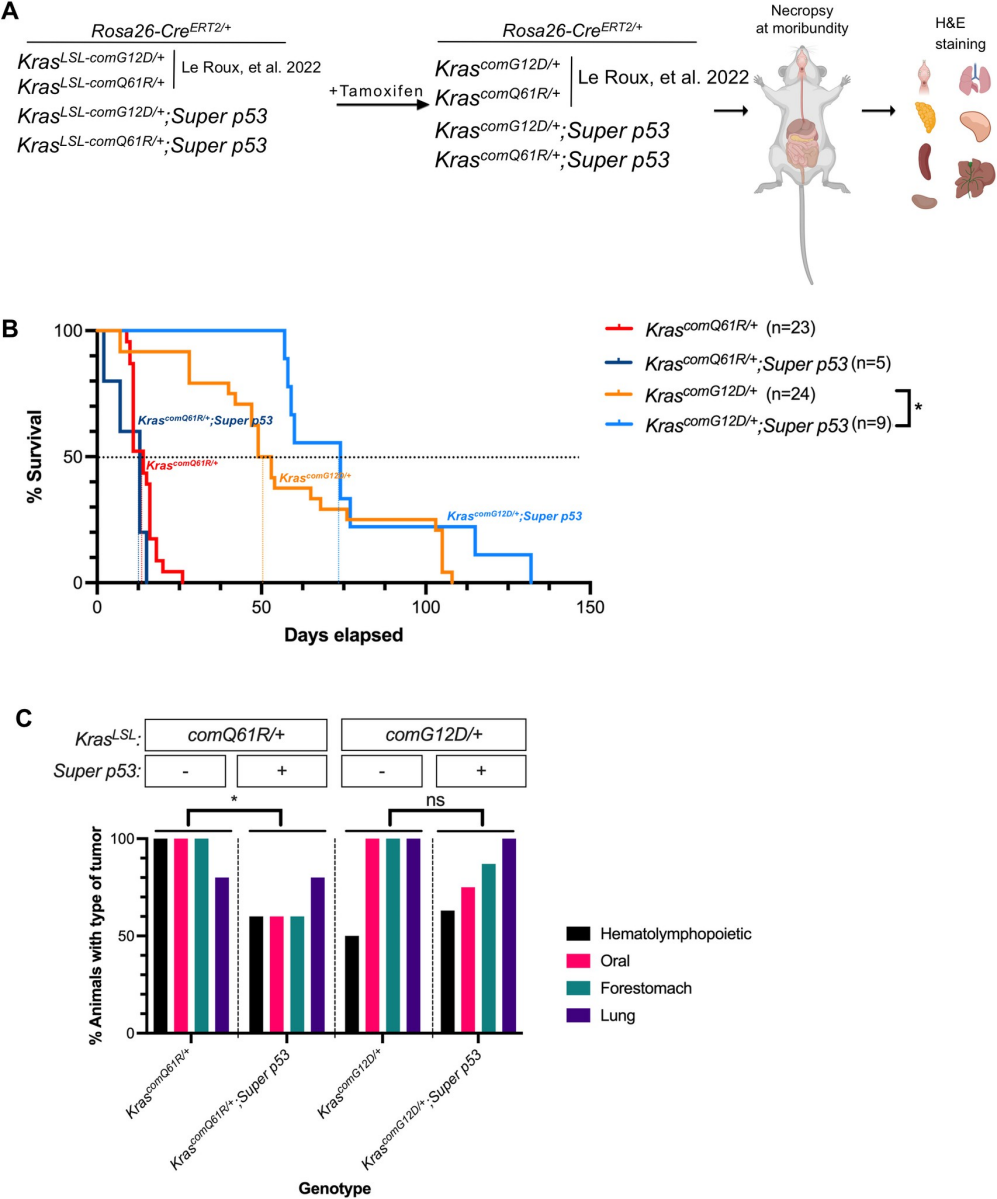
The effect of p53 dosage on Kras-driven tumorigenesis. (A) Experimental design to study the effect of the *Super p53* allele on tumorigenesis upon activating *Kras*^*LSL-comG12D*^ versus *Kras*^*LSL-comQ61R*^ alleles by tamoxifen in a *Rosa26*^*CreERT2/+*^ background. Mouse and organ images are reprinted from Biorender.com under a CC BY license, with permission from Biorender, original copyright 2023. (B) Kaplan-Meier survival curve of *Rosa26*^*CreERT2/+*^ mice with the indicated genotypes (day 0 refers to the last tamoxifen injection). Dotted lines: 50% survival. Pairwise comparison of survival curves of each genotype with versus without the *Super p53* allele were performed with Gehan-Breslow-Wilcoxon test. *: *p* = 0.047. Individual survival data values are provided in **S1 Appendix**. (C) Percent of *Rosa26*^*CreERT2/+*^ mice of the indicated genotypes (*n* = 5–8) with the described tumor types. Statistical comparisons of tumorigenesis in each genotype with and without the *Super p53* allele were done using One-way ANOVA Bonferroni’s multiple comparison test. *: *p* = 0.041. Examples of H&E-stained slides of the indicated tissues are provided in **S2 Fig.** Tumor incidence data values are provided in **S2 Appendix**.

Mice with *Rosa26*^*CreERT2/CreERT2*^ alleles were crossed with those having either of a *Kras*^*LSL-comG12D/+*^ or *Kras*^*LSL-comQ61R/+*^ genotype twice to create *Rosa26*^*CreERT2/CreERT2*^;*Kras*^*LSL-comG12D/+*^ and *Rosa26*^*CreERT2/CreERT2*^;*Kras*^*LSL-comQ61R/+*^ mice. These mice were then crossed with *Super p53* mice to create *Kras*^*LSL-comG12D/+*^;*Super p53* and *Kras*^*LSL-comQ61R/+*^;*Super p53* mice in the *Rosa26*^*CreERT2/+*^ background. Admittedly this breeding strategy yielded significantly small number of mice (around 5%) having a *Kras*^*LSL*^ background without an extra allele of *Trp53*. Thus, all tumorigenesis comparisons were made against the original tumorigenesis study in *Rosa26*^*CreERT2/CreERT2*^;*Kras*^*LSL-comG12D/+*^ and *Rosa26*^*CreERT2/CreERT2*^;*Kras*^*LSL-comQ61R/+*^ mice that lacked the extra allele of *Trp53* [10]. We generated experimental cohorts of 5 to 9 mice in *Kras*^*LSL-comG12D/+*^;*Super p53* and *Kras*^*LSL-comQ61R/+*^;*Super p53* genotypes **(S1 Fig)**, which were compared to the control cohorts of 23 to 24 mice in *Kras*^*LSL-comG12D/+*^ or *Kras*^*LSL-comQ61R/+*^ genotypes in the *Rosa26*^*CreERT2/+*^ background as previously reported [10]. All mice were injected with tamoxifen, which we previously extensively validated to result in recombination (and thus activation) of both the *Kras*^*LSL-comG12D/+*^ and *Kras*^*LSL-comQ61R/+*^ alleles in many different organs [10]. Mice were regularly monitored until moribundity, at which time they were humanely euthanized **(Fig 1A)**.

### An increase in p53 dosage provides an overall survival benefit in mice in which Kras^G12D^ is globally activated

The time of moribundity, which was previously shown to precede death due to cancer [10], was plotted for all four cohorts using the Kaplan-Meier method [15]. We previously reported the median survival of *Kras*^*comG12D/+*^ and *Kras*^*comQ61R/+*^ mice after tamoxifen injections was 14 and 51 days, respectively, in the *Rosa26*^*CreERT2/+*^ background [10]. With increased p53 dosage, the median survival was unchanged in the *Kras*^*comQ61R/+*^ cohort but increased by 23 days (51 to 74 days) in the *Kras*^*comG12D/+*^ mice, which equates to 45% increase in survival **(Fig 1B)**. Although pairwise comparisons did not show statistically significant differences between experimental and control cohorts using the Log rank test **(not shown)**, there was a statistically significant increase (*p* value <0.05) in the median survival of the *Kras*^*comG12D/+*^ mice with an extra allele of *Trp53* using the Gehan-Breslow-Wilcoxon test **(Fig 1B)**, which weighs early time points higher [16]. Thus, an extra copy of *p53* may extend lifespan in the context of the less aggressive *Kras*^*comG12D/+*^ allele.

### An increase in p53 dosage can alter tumorigenesis

To examine the effect of an increase in p53 dosage on tumorigenesis, the lung, thymus, stomach, pancreas, spleen, oral epithelia, liver, and kidney were removed from five to eight mice from each of the four cohorts and paraffin embedded and H&E stained **(Fig 1A)**. Two slides from each of these eight organs from all animals in the study were analyzed for the incidence and grading of tumors by a board-certified veterinary pathologist **(S2 Fig)**. Not surprisingly, organs previously shown to be resistant to Kras-induced tumorigenesis [10], namely the kidney, liver, and pancreas, remained tumor-free **(not shown)**. In terms of the percentage of mice with tumors in organs in which tumors were detected, namely hematolymphopoietic, oral, forestomach and lung tissues, there was a statistically significant decrease in *Kras*^*comQ61R/+*^, but not *Kras*^*comG12D/+*^ mice in the *Super p53* background **(Fig 1C)**.

### Oral and forestomach squamous epithelial lesions

**Fig 2:**
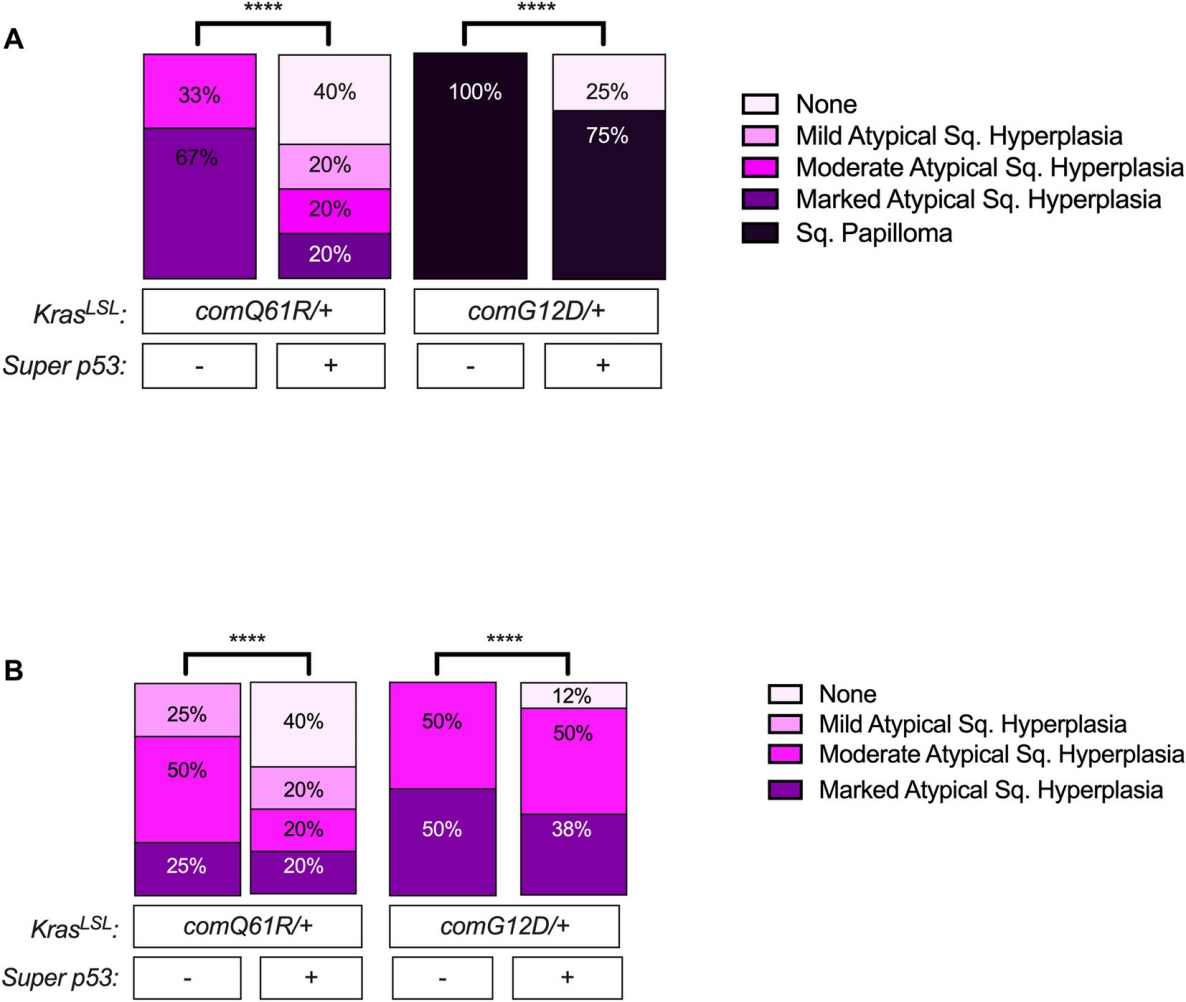
The effect of p53 dosage on Kras-driven oral and forestomach tumorigenesis. Percentage of *Rosa26*^*CreERT2/+*^ mice of the indicated genotypes (*n* = 5–8) with the indicated grades of (A) oral and (B) forestomach squamous epithelial lesions at moribundity endpoint. Pairwise comparisons of severity of oral and forestomach lesions in each genotype with versus without *Super p53* were performed using Chi-square test. ****: *p*-value <0.0001. Oral and forestomach squamous epithelial lesion incidence and grading data values are provided in **S3 Appendix**.

We previously reported that oral and forestomach squamous epithelial lesions were more prevalent and aggressive in a *Kras*^*LSL-comG12D/+*^ compared to a *Kras*^*LSL-comQ61R/+*^ background, suggesting that these two tissues were particularly sensitive to the ability of the G12D mutant of Kras to induce tumorigenesis [10]. To explore the impact of an extra allele of *Trp53* on the tumorigenesis in these two tissues, the aforementioned H&E-stained slides were analyzed and graded as either mild, moderate, or marked atypical squamous hyperplasia (ASH) or as squamous papilloma. With increased p53 dosage, the incidence of the oral lesions significantly decreased by 40% in the *Kras*^*comQ61R/+*^ and by 25% in the *Kras*^*comG12D/+*^ background, while the severity of the oral squamous epithelial lesions were reduced in both the *Kras*^*comG12D/+*^ and *Kras*^*comQ61R/+*^ background **(Fig 2A)**. Namely, the percentage of mice with the *Kras*^*comG12D/+*^ alleles that had the most severely graded oral lesions of squamous papilloma decreased from 100% to 75% with an extra copy of *Trp53*, while there was a clear shift from more severe lesions of marked and moderate ASH to less severe lesions of mild ASH or no lesions in the *Kras*^*comQ61R/+*^ background with increased p53 dosage **(Fig 2A)**. The effect of increased p53 dosage on the incidence and severity of lesions was similar in forestomach squamous epithelial lesions, although less pronounced **(Fig 2B)**. Specifically, with increased p53 dosage, the percentage of animals with forestomach squamous epithelial lesions decreased in *Kras*^*comQ61R/+*^ and *Kras*^*comG12D/+*^ background by 40% and 13%, respectively. Furthermore, in both *Kras*^*comQ61R/+*^ and *Kras*^*comG12D/+*^ backgrounds, increasing p53 dosage led to a shift from more severe to less severe squamous epithelial lesion types in the forestomach, which was more noticeable in the *Kras*^*comQ61R/+*^ genotype **(Fig 2B)**. Thus, the *Super p53* background reduced oral and forestomach squamous epithelial tumorigenesis induced by either Kras-mutant allele.

### Hematolymphopoietic lesions

**Fig 3:**
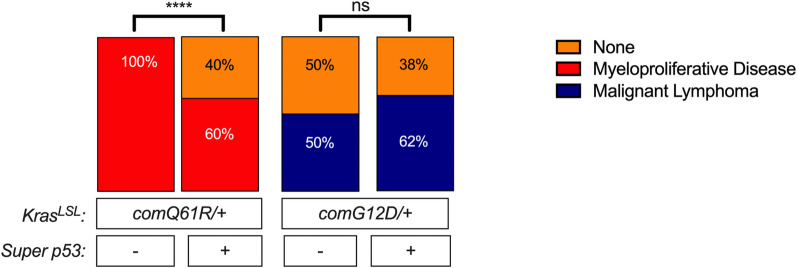
The effect of p53 dosage on Kras-driven hematolymphopoietic tumorigenesis. Percent of *Rosa26*^*CreERT2/+*^ mice of the indicated genotypes (*n* = 5–8) with the indicated type of hematolymphopoietic lesions of myeloproliferative disease and lymphomas. Pairwise comparisons of hematolymphopoietic tumor incidence in each genotype with versus without *Super p53* were performed using Chi-square test. ****: *p*-value <0.0001. Data values of hematolymphopoietic tumor incidence and presence of hematolymphopoietic infiltrates in different tissues are provided in **S4 Appendix**.

We previously reported that hematolymphopoietic disease was more severe either through increased Kras expression or in the *Kras*^*comQ61R/+*^ mutant background, suggesting that this tissue was particularly sensitive to the level of active Kras to induce tumorigenesis [10]. Given this, we compared the number of mice with myeloproliferative disease as well as the number and grade of mice with lymphomas. Specifically, the aforementioned H&E slides of all tissues from each of the four genotypes were analyzed for the presence of hematolymphopoietic lesions. As previously reported, the myeloproliferative disease was only detected in mice with Q61R mutation [10] and the percentage of animals with myeloproliferative disease decreased from 100% to 60% with an extra copy of *Trp53* in this background **(Fig 3)**. Consistent with decreased incidence, percentage of mice having the *Kras*^*comQ61R/+*^ genotype with myeloproliferative infiltrates in spleen, kidney, liver, pancreas, thymus, and lung was decreased with increased p53 dosage **(S3A Fig)**. When we compared the impact of the extra *Trp53* allele on G12D-induced hematolymphopoietic lesions, we observed that the percentage of animals with lymphoma showed a slight increase, although this change was not statistically significant **(Fig 3)**. Increasing p53 dosage in *Kras*^*comG12D/+*^ genotype altered the lymphoma infiltration to different organs. Namely, lymphoma infiltration to the thymus, spleen, and lung was increased, infiltration to the kidney and liver was decreased, while infiltration was absent in the pancreas **(S3B Fig)**. Thus, an additional copy of *Trp53* appears to suppress myeloproliferative disease in the *Kras*^*comQ61R/+*^ background.

### Lung lesions

**Fig 4:**
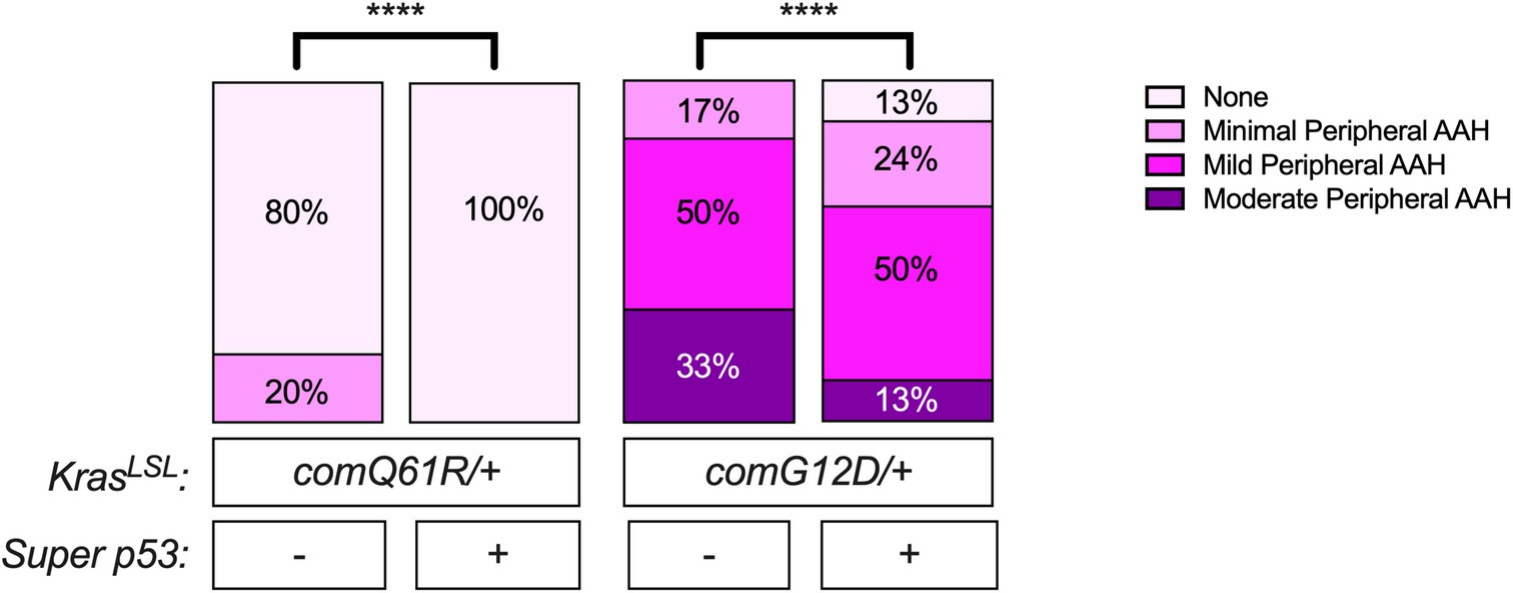
The effect of p53 dosage on Kras-driven lung tumorigenesis. Percentage of *Rosa26*^*CreERT2/+*^ mice with the indicated grades of peripheral atypical alveolar hyperplasia (AAH) detected in each genotype (*n* = 5–8). Statistical comparisons of the severity of peripheral AAH in each genotype with versus without *Super p53* allele were done using Chi-square test. ****: *p*-value <0.0001. The incidence, grading, and number of lesions in the lung are provided in **S5 Appendix**.

Although the percentage of animals with lung lesions did not change in both *Kras*^*comQ61R/+*^ and *Kras*^*comG12D/+*^ mice **(Fig 1C)**, upon comparing the grading of the lesions in the lungs, we found that with increased p53 dosage, the severity of the peripheral atypical alveolar hyperplasia (AAH) was reduced in both *Kras*^*comQ61R/+*^ and *Kras*^*comG12D/+*^ mice. Specifically, with an extra copy of *Trp53*, the percentage of animals with peripheral AAH was reduced from 20% to 0% in the *Kras*^*comQ61R/+*^ background and from 100% to 87% in the *Kras*^*comG12D/+*^ background **(Fig 4)**. Interestingly, the number of *Kras*^*comQ61R/+*^-induced bronchioloalveolar hyperplasia (BAH) foci was not changed, with the caveat that the myeloproliferative infiltration in *Kras*^*comQ61R/+*^ background precluding accurate determination of the number of lung lesions. However, there was a significant increase in the number of BAH foci in the *Kras*^*comG12D/+*^ mice with increased p53 dosage **(S4A Fig)**. On the other hand, the number of adenomas was not changed in both *Kras*^*comG12D/+*^ and *Kras*^*comQ61R/+*^ backgrounds **(S4B Fig)**. While admittedly spectulative, since both alveolar type II and bronchiolar club cells are known to initiate tumors that may progress to adenomas [17], perhaps the unchanged level of adenomas is a product of increased in BAH lesions in spite of a reduced number of peripheral atypical alveolar lesions **(S4A and S4B Fig)**.

## Discussion

Increasing p53 dosage extended lifespan in *Rosa26*^*CreERT2/+*^*;Kras*^*LSL-comG12D/+*^ mice by one statistical comparison, and this was associated with a decrease in the incidence and/or grade of squamous lesions of the oral and forestomach epithelia. This extension of lifespan was not observed in the *Rosa26*^*CreERT2/+*^*;Kras*^*LSL-comQ61R/+*^ mice upon increasing p53 dosage, although the percentage of these mice with lesions in the oral cavity, forestomach, and hematolymphopoietic compartment decreased, and peripheral AAH in the lung was diminished. This discordance between no change in lifespan but a reduction in some forms of tumorigenesis could be a product of two effects. First, activating the *Kras*^*comQ61R*^ allele leads to moribundity endpoints in 13 to 14 days, which is incredibly fast, ostensibly due to myeloproliferative disease. Thus, a reduction in myeloproliferative disease may simply not be enough to change survival. Further, since bone marrow was not collected in the original study design, we cannot exclude the possibility of reduced bone marrow function even in the presence of an extra copy of the *Trp53* gene. Second, we observed a significant decrease in only squamous tumors with increased p53 dosage in the *Kras*^*comG212D/+*^ background. The most common reason for moribundity in this cohort was either oral tumors reaching maximum humane size, ulcerated oral tumors, or sudden loss of weight [10]. Since an additional copy of the *Trp53* gene decreased the percent incidence and grade of these squamous tumors, the survival benefit is presumably a product decrease in squamous tumorigenesis in oral and/or stomach epithelia, which could be explained as benefit from better food intake and nutrition for a slightly longer period of time. Nevertheless, the actual reasons for why the additional copy of *Trp53* altered tumorigenesis without extending lifespan in the *Kras*^*comQ61R/+*^ background remains to be elucidated.

An extra copy of *Trp53* did not always suppress tumorigenesis. For example, the percentage of animals with lung tumors in both *Kras*^*comQ61R/+*^ and *Kras*^*comG12D/+*^ genotypes, and the hematolymphopoietic cancers in the *Kras*^*comG12D/+*^ genotype did not change with increased p53 dosage **(Figs 1C and 3)**. Similarly, *Kras*^*comQ61R/+*^ and *Kras*^*comG12D/+*^ induced similar number of adenomas per animal with and without the extra allele of *Trp53* **(S4B Fig)**. However, with increased p53 dosage, we observed a significant increase in the number of BAH foci **(S4A Fig)** and 13% decrease in percentage of animals showing peripheral AAH in the lung **(Fig 4)** in the *Kras*^*comG12D/+*^ genotype, perhaps reflecting a decrease in progression of BAH to adenomas.

We note the following caveats to this study. First, due to the breeding strategy, the number of littermates with the control genotypes *Rosa26*^*CreERT2/+*^;*Kras*^*LSL-comG12D/+*^ or *Rosa26*^*CreERT2/+*^;*Kras*^*LSL-comQ61R/+*^ was extremely limited, and thus we chose to make comparisons to the control genotypes reported in our previous study as all experimental steps were performed in the same manner. As such, some trends may be the product of the natural variation in tumorigenesis rather than a bona fide phenotypic difference. However, the reduction in the number and/or grade of tumors was a reoccurring theme in the *Super p53* background, supporting this as a real phenomenon. Second, we have not measured the level of p53 protein in the *Trp53*^*+/+*^ versus *Super p53* backgrounds to confirm an increase in the latter is linked to the observed phenotypes. However, by the very nature of providing an extra copy of this gene, p53 protein was previously demonstrated to increase with genotoxic insult in mouse embryonic fibroblasts with subsequent decrease in cell cycle arrest [12]. Third, we appreciate that the *Kras*^*LSL-comG12D/+*^ and *Kras*^*LSL-comQ61R/+*^ alleles have altered the normal structure of the *Kras* gene, and hence limit the degree that these two alleles can be compared to other modified *Kras* alleles. Nevertheless, by using two alleles constructed in the identical fashion, meaningful comparisons could be made.

In summary, with these exceptions and caveats, the extra copy of *Trp53* appears to be generally suppressive in squamous tumors of oral and forestomach epithelia regardless of the type of oncogenic Kras mutation used to initiate tumorigenesis, although the effect was more pronounced when tumorigenesis was less aggressive. While admittedly very speculative, we suggest that given these findings, perhaps then naturally occurring variations in the level of p53 protein in the human population may influence the likelihood that a spontaneously arising KRAS mutation initiates tumorigenesis, which may find value in predicting susceptibility to KRAS-driven cancers or in development of therapeutic strategies against KRAS-induced cancers.

## Materials and methods

### Mouse strains

*Kras*^*LSL-comG12D/+*^ and *Kras*^*LSL-comQ61R/+*^ mice were previously described and were from a pure 129 background [10]. *Super p53* mice [12] were kindly provided by David Kirsch (University of Toronto) and were from C57BL/6 background. *Rosa26*^*CreERT2/CreERT2*^ mice were obtained from The Jackson Laboratory (strain #008463). All animals derived in subsequent crosses were selected by genotyping using the genotyping methods previously described [10, 12].

### Tumorigenesis studies

*Kras*^*LSL-comG12D/+*^ and *Kras*^*LSL-comQ61R/+*^ mice were crossed with mice with *Rosa26*^*CreERT2/CreERT2*^ allele (Jackson Laboratory, strain 008463) to generate *Rosa26*^*CreERT2/+*^;*Kras*^*LSL-comG12D/+*^ and *Rosa26*^*CreERT2/+*^;*Kras*^*LSL-comQ61R/+*^ mice. These *Kras*^*LSL*^ mice in the *Rosa26*^*CreERT2/+*^ background were then crossed with the *Rosa26*^*CreERT2/CreERT2*^ mice again to generate *Rosa26*^*CreERT2/CreERT2*^;*Kras*^*LSL-comG12D/+*^ and *Rosa26*^*CreERT2/CreERT2*^;*Kras*^*LSL-comQ61R/+*^ mice. *Kras*^*LSL*^ mice with homozygous Cre alleles were then crossed with the *Super p53* mice to generate the littermates of *Kras*^*LSL-comG12D/+*^ and *Kras*^*LSL-comG12D/+*^;*Super p53*, and *Kras*^*LSL-comQ61R/+*^ and *Kras*^*LSL-comQ61R/+*^;*Super p53* mice in the *Rosa26*^*CreERT2/+*^ background. This crossing strategy generated *Kras*^*+/+*^*;Super p53* mice at the ratio of 80%, leading to relatively small number of *super p53* mice harboring a *Kras*^*LSL*^ allele in the *Rosa26*^*CreERT2/+*^ background (around 10%), while the ratio of littermates with the *Kras*^*LSL*^ allele in the *Rosa26*^*CreERT2/+*^ background without the extra *Trp53* was around 5% **(S1 Fig)**. Despite our efforts to compare littermates of *Kras*^*LSL*^ mice with and without an extra allele of *Trp53*, with all littermates in the *Rosa26*^*CreERT2/+*^ background, this strategy came at the cost of yielding significantly small number of control littermates with a *Kras*^*LSL*^ background without an extra allele of *Trp53* after multiple rounds of crosses. Using this strategy, we generated experimental cohorts of 7 to 9 mice with random distribution of males and females in *Kras*^*LSL-comG12D/+*^;*Super p53* and *Kras*^*LSL-comQ61R/+*^;*Super p53* genotypes. For comparison of median survival, comparisons were made against the control cohorts we reported previously with 23 to 24 mice with random distribution of males and females in *Kras*^*LSL-comG12D/+*^ or *Kras*^*LSL-comQ61R/+*^ genotypes in the *Rosa26*^*CreERT2/+*^ background [10]. For comparison of tumorigenesis, we chose to limit the number of control animals in the study to a similar number in the experimental cohort to make statistical power evenly distributed across cohorts. Thus, tumorigenesis comparisons were made against 5 to 6 randomly chosen mice from the control cohorts with random distribution of males and females in *Kras*^*LSL-comQ61R/+*^ and *Kras*^*LSL-comG12D/+*^ genotypes in the *Rosa26*^*CreERT2/+*^ background [10]. Tumorigenesis studies were performed in the same manner as reported previously **(Fig 1A)** [10]. Namely, tamoxifen (Sigma-Aldrich, T5648-5G, CAS# 10540-29-1) was dissolved in corn oil (Sigma-Aldrich, C8267) and filter sterilized. At six to eight weeks of age, experimental cohorts of 7 to 9 mice with random distribution of males and females in *Kras*^*LSL-comG12D/+*^;*Super p53* and *Kras*^*LSL-comQ61R/+*^;*Super p53* genotypes were injected intraperitoneally with 250 μg/g body weight of tamoxifen four times every 48 hrs. Tamoxifen injections were performed under anesthesia by isoflurane to prevent suffering. After recombination of the *LSL* cassette with tamoxifen, mice were observed daily during injections, 1 week after last injection, and weekly thereafter. Mice were humanely euthanized with carbon dioxide inhalation followed by removal of vital organs within 2 hrs of detection of moribundity humane endpoint. To prevent suffering and pain, we defined moribundity humane endpoint as any of the visible signs of sudden behavioral change, poor/hunched posture, lost hair coat condition, sudden activity level change, painful facial expression, signs of pain that was not anticipated by the study plan, weight loss of exceeding 15% compared to an age-matched reference, and cardiopulmonary disorders. Humane euthanasia was performed via CO_2_ inhalation followed by removal of essential organs as a secondary method. Selected tissues, including lung, thymus, stomach, pancreas, spleen, oral epithelia, liver, and kidney, were removed at necropsy followed by fixation in 10% formalin (VWR, 89370–094) for 24–48 hrs, then were stored in 70% ethanol (VWR, 89125–166) until routine processing. Two animals from the *Kras*^*LSL-comQ61R/+*^;*Super p53* genotype were found dead within 24 hrs of the first tamoxifen injection, thus, were not included in either of the reported survival or tumorigenesis studies as we could not perform necropsy in a timely manner. Tissues were sliced with one-to-two-millimeter thickness. Tissue slices were embedded in paraffin with the flat sides down, sectioned at a depth of 5 μm, and stained by the H&E method by IDEXX Laboratories. All H&E slides were evaluated by a board-certified veterinary pathologist with experience in murine pathology in a blinded fashion.

### Statistical analysis

Statistical analyses were performed using GraphPad Prism software version 9.5.1 (GraphPad Software). Pairwise comparisons of the survival plots of each cohort with and without an extra allele of *Trp53* were performed with Log-rank (Mantel-Cox) and Gehan-Breslaw-Wilcoxon test [18] **(**not shown and **Fig 1B)**. Statistical comparisons of percentage of mice with detected tumors in different organs with and without *Super p53* allele were done using One-way ANOVA Bonferroni’s multiple comparison test with 95% confidence interval (CI) **(Fig 1C)**. Pairwise comparisons of oral, forestomach, and peripheral AAH lesions, and hematolymphopoietic diseases with and without *Super p53* were performed using Chi-square test **(Figs 2A, 2B, 3 and 4)**. For comparisons of number of foci of BAH lesions and adenomas per animal with versus without *Super p53*, one-way ANOVA with Bonferroni’s multiple-comparisons test with a single pooled variance and a 95% CI were used **(S4A and S4B Fig)**. A *p*-value of less than 0.05 was considered statistically significant.

### Ethics statement

This study was carried out in strict accordance with the recommendations in the Guide for the Care and Use of Laboratory Animals of the National Institutes of Health. All animal experiments were approved in writing by Duke University Institutional Animal Care and Use Committee (IACUC) with protocol number A143-22-08. All injections were performed under isoflurane anesthesia and all efforts were made to minimize suffering. To prevent suffering and pain during tumorigenesis study, we defined moribundity humane endpoint as any of the visible signs of sudden behavioral change, poor/hunched posture, lost hair coat condition, sudden activity level change, painful facial expression, signs of pain that was not anticipated by the study plan, weight loss of exceeding 15% compared to an age-matched reference, and cardiopulmonary disorders. Humane euthanasia was performed via CO_2_ inhalation followed by removal of essential organs as a secondary method.

## Supporting information

S1 Fig Breeding strategy to generate the indicated genotypes. Mouse images are reprinted from Biorender.com under a CC BY license, with permission from Biorender, original copyright 2023.
(TIF)

S2 Fig Examples of lesions identified in H&E-stained tissues. Examples of lesions identified in H&E staining of the indicated organs removed at moribundity endpoint from *Rosa26*^*CreERT2/+*^ mice with the four indicated genotypes. Higher magnification of H&E-stained sections of liver are depicted with black rectangles. Arrows point to the examples of peripheral atypical alveolar hyperplasia (AAH), bronchioloalveolar hyperplasia (BAH), and pulmonary adenoma, and hematolymphopoietic infiltrates in the liver from each genotype. Scale bars are provided. H&E-stained images of the indicated organs from *Rosa26*^*CreERT2/+*^ mice with *Kras*^*LSL-comQ61R*^ or *Kras*^*LSL-comG12D*^ alleles in the absence of the *Super p53* allele are novel but the samples were derived from a previous study [10].
(TIF)

S3 Fig Hematolymphopoietic infiltrates. (A,B) Percent of *Rosa26*^*CreERT2/+*^ mice (*n* = 5–8) with hematolymphopoietic infiltrates in the indicated organs upon activating the (A) *Kras*^*LSL-comQ61R*^ or (B) *Kras*^*LSL-comG12D*^ alleles in the absence and presence of the *Super p53* allele. (C) Examples showing lymphoma infiltrates in an H&E-stained section of the indicated organs from a *Rosa26*^*CreERT2/+*^*;Kras*^*LSL-comG12D*^ versus *Rosa26*^*CreERT2/+*^*;Kras*^*LSL-comG12D*^*;Super p53* mouse.
(TIF)

S4 Fig The effect of p53 dosage on the types of Kras-driven lung tumorigenesis. Number of lung (A) bronchioloalveolar hyperplasia foci or (B) adenomas per animal in *Rosa26*^*CreERT2/+*^ mice of the indicated genotypes (*n* = 5–8). *: mice with extensive myeloid infiltrates that precludes accurate determination of the number of lung lesions. One-way ANOVA with Bonferroni’s multiple-comparisons test with a single pooled variance and a 95% CI were used to identify the significance of the effect of an extra allele of *Trp53*. **: *p* = 0.002.
(TIF)

S1 Appendix Individual survival data values. (XLSX)

S2 Appendix Percent animals with different types of tumors data values. (XLSX)

S3 Appendix Oral and forestomach squamous epithelial lesion incidence and grading data values. (XLSX)

S4 Appendix Hematolymphopoietic cancer incidence and infiltrate data values. (XLSX)

S5 Appendix Incidence, grading, and number of lung lesions. (XLSX)
