# Supplementary figures and images for: p53 dosage can impede KrasG12D- and KrasQ61R-mediated tumorigenesis
Source: PLoS One. 2024 Mar 28;19(3):e0292189. doi: 10.1371/journal.pone.0292189 (PMC10977719; doi:10.1371/journal.pone.0292189)

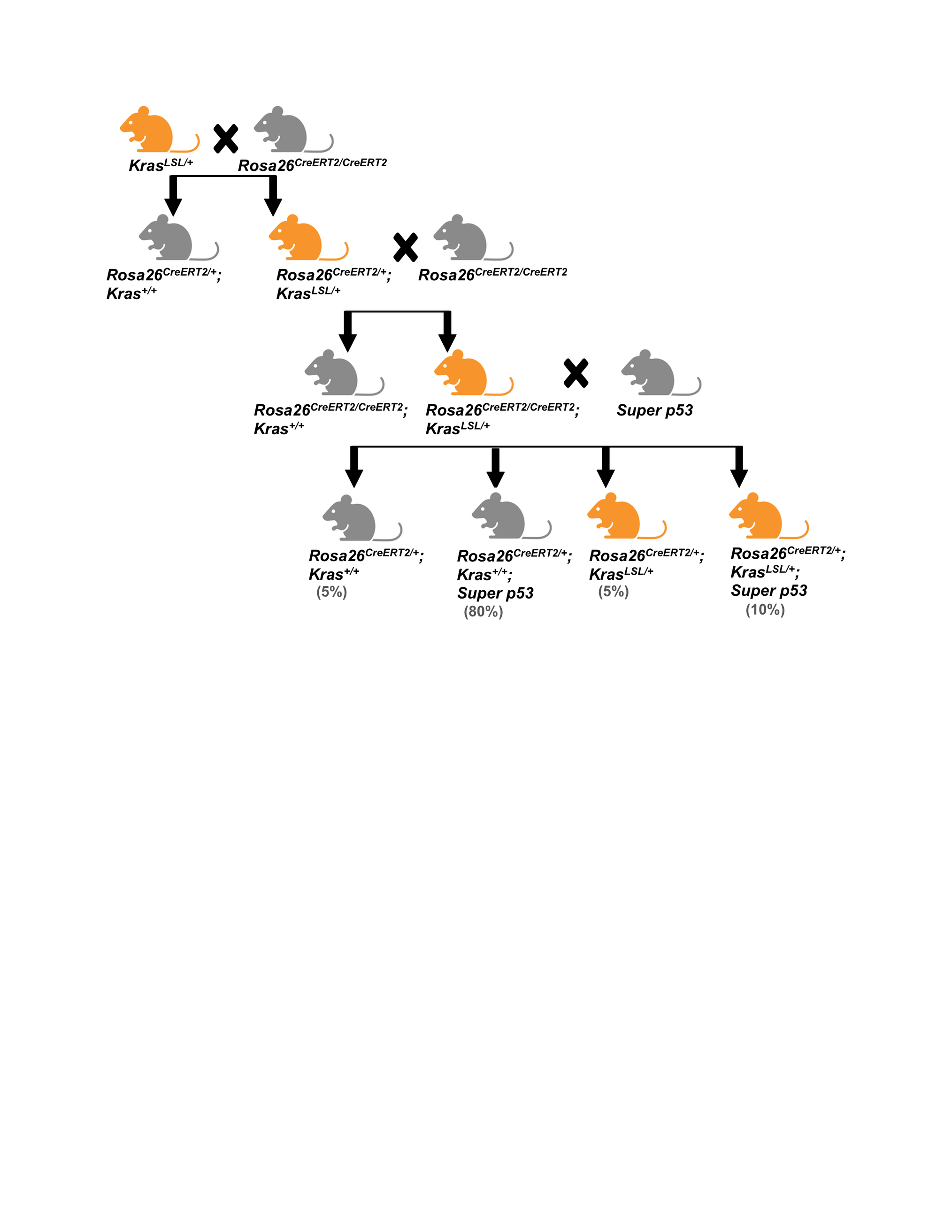

Supplement: S1 Fig — Mouse images are reprinted from Biorender.com under a CC BY license, with permission from Biorender, original copyright 2023. (TIF) [file pone.0292189.s001.tif]

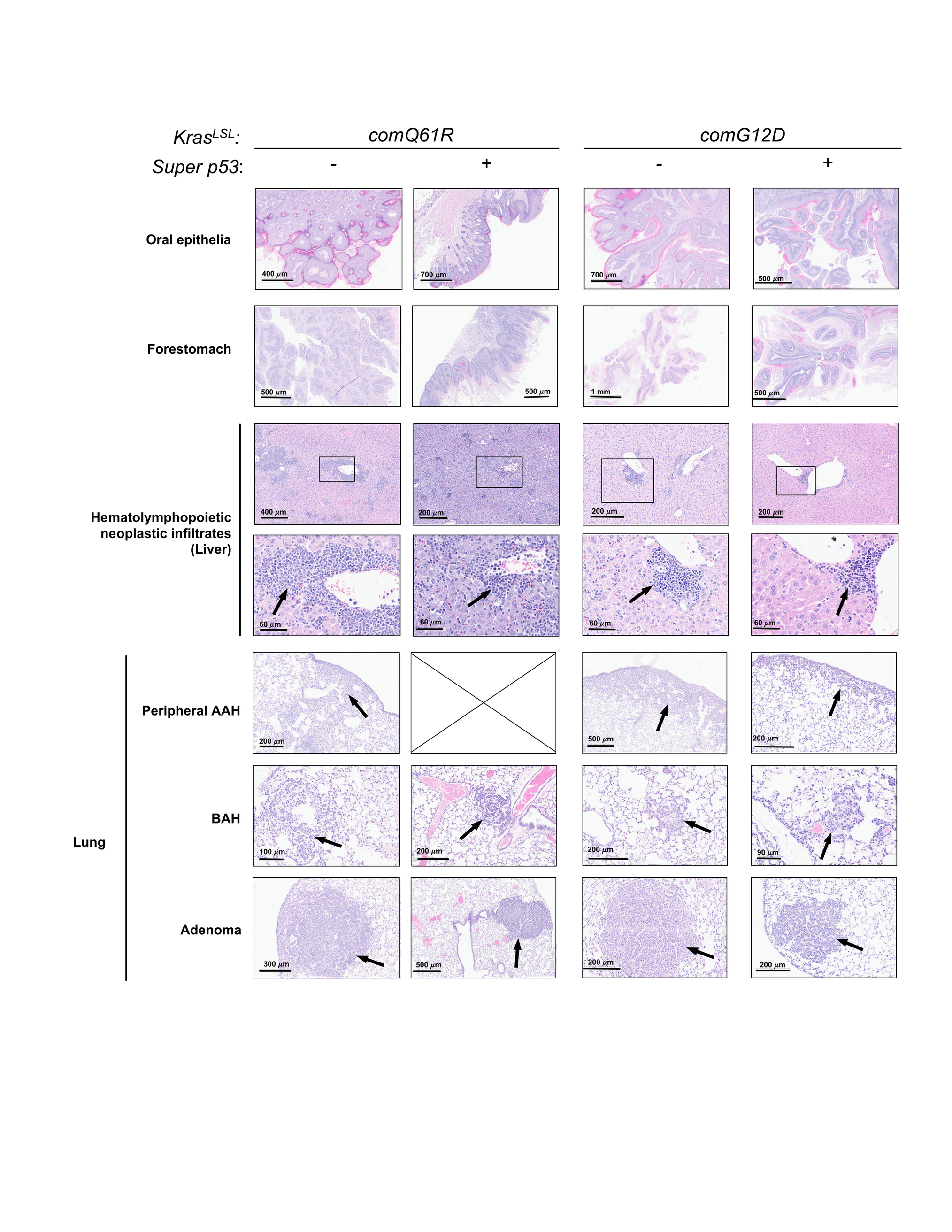

Supplement: S2 Fig — Examples of lesions identified in H&E staining of the indicated organs removed at moribundity endpoint from Rosa26CreERT2/+ mice with the four indicated genotypes. Higher magnification of H&E-stained sections of liver are depicted with black rectangles. Arrows point to the examples of peripheral atypical alveolar hyperplasia (AAH), bronchioloalveolar hyperplasia (BAH), and pulmonary adenoma, and hematolymphopoietic infiltrates in the liver from each genotype. Scale bars are provided. H&E-stained images of the indicated organs from Rosa26CreERT2/+ mice with KrasLSL-comQ61R or KrasLSL-comG12D alleles in the absence of the Super p53 allele are novel but the samples were derived from a previous study [10]. (TIF) [file pone.0292189.s002.tif]

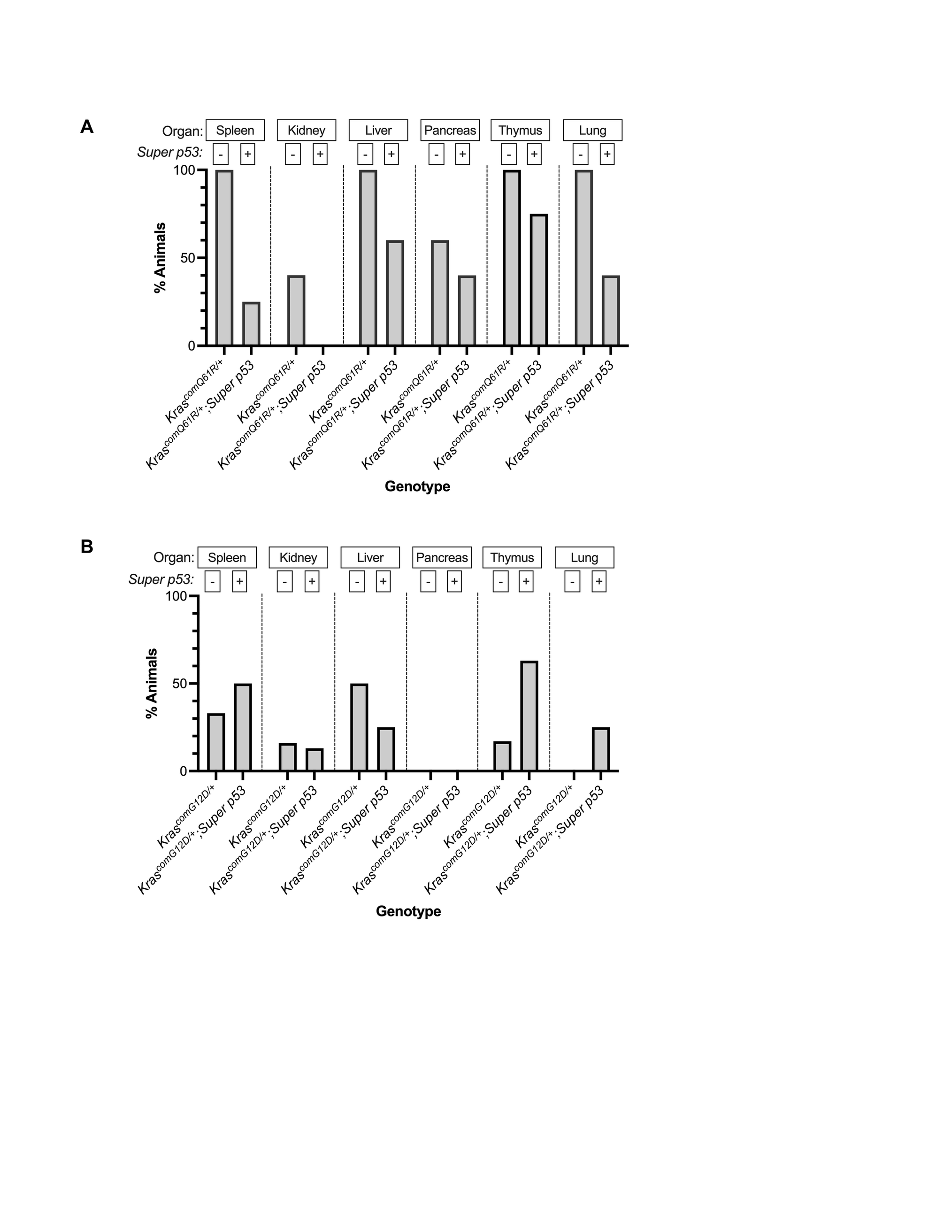

Supplement: S3 Fig — (A,B) Percent of Rosa26CreERT2/+ mice (n = 5–8) with hematolymphopoietic infiltrates in the indicated organs upon activating the (A) KrasLSL-comQ61R or (B) KrasLSL-comG12D alleles in the absence and presence of the Super p53 allele. (C) Examples showing lymphoma infiltrates in an H&E-stained section of the indicated organs from a Rosa26CreERT2/+;KrasLSL-comG12D versus Rosa26CreERT2/+;KrasLSL-comG12D;Super p53 mouse. (TIF) [file pone.0292189.s003.tif]

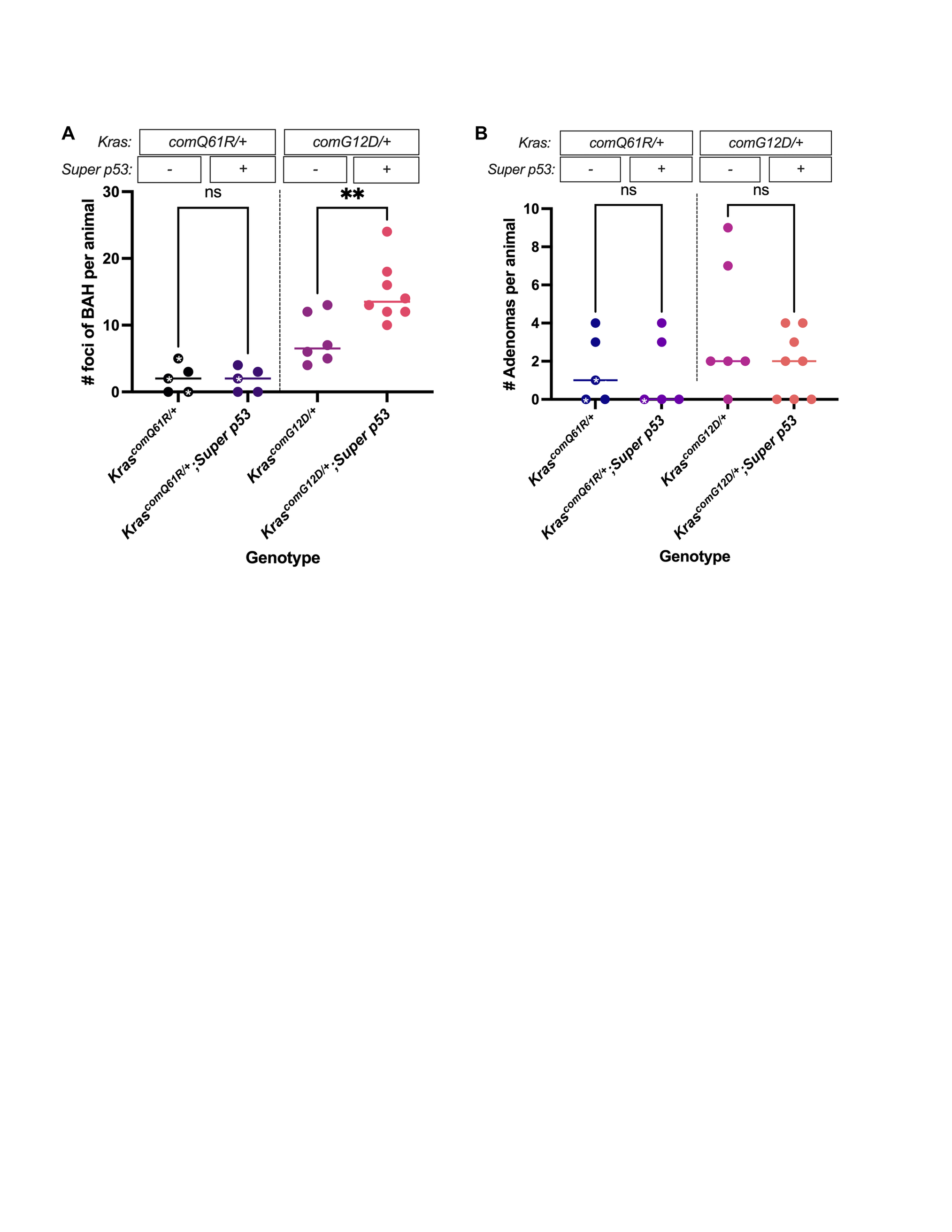

Supplement: S4 Fig — Number of lung (A) bronchioloalveolar hyperplasia foci or (B) adenomas per animal in Rosa26CreERT2/+ mice of the indicated genotypes (n = 5–8). *: mice with extensive myeloid infiltrates that precludes accurate determination of the number of lung lesions. One-way ANOVA with Bonferroni’s multiple-comparisons test with a single pooled variance and a 95% CI were used to identify the significance of the effect of an extra allele of Trp53. **: p = 0.002. (TIF) [file pone.0292189.s004.tif]
